# Long non-coding RNA (LncRNA) and epigenetic factors: their role in regulating the adipocytes in bovine

**DOI:** 10.3389/fgene.2024.1405588

**Published:** 2024-10-03

**Authors:** Diba Dedacha Jilo, Belete Kuraz Abebe, Jianfang Wang, Juntao Guo, Anning Li, Linsen Zan

**Affiliations:** ^1^ College of Animal Science and Technology, Northwest A&F University, Yangling, Shaanxi, China; ^2^ Department of Animal Science, Bule Hora University, Bule Hora, Ethiopia; ^3^ Department of Animal Science, Werabe University, Werabe, Ethiopia; ^4^ National Beef Cattle Improvement Center, Northwest A&F University, Yangling, Shaanxi, China

**Keywords:** bovine adipocytes, epigenetic factors, lncRNAs function, regulation role, meat quality

## Abstract

Investigating the involvement of long non-coding RNAs (lncRNAs) and epigenetic processes in bovine adipocytes can provide valuable new insights into controlling adipogenesis in livestock. Long non-coding RNAs have been associated with forming chromatin loops that facilitate enhancer-promoter interactions during adipogenesis, as well as regulating important adipogenic transcription factors like C/EBPα and PPARγ. They significantly influence gene expression regulation at the post-transcriptional level and are extensively researched for their diverse roles in cellular functions. Epigenetic modifications such as chromatin reorganization, histone alterations, and DNA methylation subsequently affect the activation of genes related to adipogenesis and the progression of adipocyte differentiation. By investigating how fat deposition is epigenetically regulated in beef cattle, scientists aim to unravel molecular mechanisms, identify key regulatory genes and pathways, and develop targeted strategies for modifying fat deposition to enhance desirable traits such as marbling and meat tenderness. This review paper delves into lncRNAs and epigenetic factors and their role in regulating bovine adipocytes while focusing on their potential as targets for genetic improvement to increase production efficiency. Recent genomics advancements, including molecular markers and genetic variations, can boost animal productivity, meeting global demands for high-quality meat products. This review establishes a foundation for future research on understanding regulatory networks linked to lncRNAs and epigenetic changes, contributing to both scholarly knowledge advancement and practical applications within animal agriculture.

## 1 Introduction

The highly regulated process of adipocyte differentiation, which is the transition of precursor cells into adipocytes, is essential for preserving energy balance and adipose tissue formation (Kang et al., 2020; Squillaro et al., 2020; Zhang R et al., 2021). The complex biological process leading to the growth of adipocytes is regulated by transcription factors, communication channels, hormones, and other entities (Liu et al., 2020; Li J et al., 2022). The Wnt (wingless-type MMTV integration site) signaling pathway regulates the adipogenic process through both conventional (dependent) and non-conventional (independent) mechanisms (Wang et al., 2020; Liu et al., 2020). The initiation of preadipocyte growth is overseen by the stimulation of enzymes through the MAPK (mitogen-activated protein kinases) signaling chain (Kang et al., 2020; Zhang W et al., 2023). Adipogenesis is the process by which preadipocytes differentiate into mature adipocytes. It is a highly regulated biological mechanism that has attracted a lot of study interest. Following this, the preadipocytes go through a process known as terminal differentiation, which is marked by the expression of genes unique to adipocytes as well as the acquisition of the normal morphological and functional traits of mature adipocytes (Wang L et al., 2021). The growth and remodeling of adipose tissue in response to shifts in energy balance depends on the proliferation and differentiation of adipocytes. Preadipocytes can differentiate into adipocytes and are found in the stromal-vascular component of adipose tissue. A complex network of transcriptional regulators, signaling channels, and epigenetic alterations works together to manage the well-planned process of adipocyte development (Squillaro et al., 2020). A great resource for understanding the molecular mechanisms behind preadipocyte development is the mouse 3T3-L1 cell line, which is the most extensively researched model for adipogenesis. Multistep and strictly controlled by a series of transcription factors, such as CCAAT/enhancer-binding proteins, is the process by which preadipocytes differentiate into mature adipocytes (Li et al., 2023).

The peroxisome proliferator-activated receptor (PPAR), which links the promoter’s reaction components, controls the transcription of target genes. The elements of the promoter’s response encompass the various molecules and entities involved in overseeing gene expression (Li Z et al., 2022). These constituents comprise transcription factors, coactivators or corepressors, DNA-binding proteins, and other regulatory components. The peroxisome proliferator-activated receptor consolidates these elements and manages their interactions to govern the activation of specific genes (Tripathi and Walker, 2016). Through examination of three-dimensional crystal structures, it has been discovered that PPARs possess larger and more accessible ligand-binding pockets compared to other nuclear receptors. This characteristic enables PPARs to attach to a wide array of substances such as fatty acids and metabolites, ultimately resulting in their stimulation and regulation of metabolic processes (Zoete et al., 2007). Through CCAAT/enhancer-binding protein (CEBP), genes are transcriptionally controlled to promote differentiation (Hu et al., 2023).

Epigenetic factors and lncRNAs have become crucial regulators in this intricate process. These regulatory elements work together to adjust gene expression and ultimately determine the destiny of precursor cells as adipocytes (Zeng et al., 2018; Sun and Lin, 2019). Their interactions impact significant processes like chromatin remodeling, Histone changes and DNA methylation subsequently impact the activation of adipogenesis-related genes and the advancement of adipocyte differentiation (Mercer and Mattick, 2013; Wei, et al., 2016; Squillaro et al., 2020). It is essential to comprehend how lncRNAs and epigenetic factors interact for understanding the molecular processes involved in adipocyte differentiation, which could have implications for developing treatments to regulate adipogenesis and address obesity (Chen, 2016; Scheideler, 2019). Furthermore, investigating the interaction between lncRNAs and epigenetics in bovine adipocytes can provide important new understandings into the control of adipogenesis in livestock (Shi et al., 2019; Zhang S et al., 2021; Xiao et al., 2021). Through regulating gene expression at the transcriptional and post-transcriptional levels, these lncRNAs have been found to affect a number of cellular processes, including lipid metabolism and adipocyte differentiation (Hu et al., 2023). Moreover, lncRNAs have been linked to the development of chromatin loops that promote enhancer-promoter interactions during adipogenesis and the regulation of significant adipogenic transcription factors, such as C/EBPα and PPARγ (Zhao et al., 2020; Ma X et al., 2023).

Enhancing the efficiency of beef production and comprehending the growth of fat cells in beef cattle require a thorough grasp of the functions and molecular mechanisms of lncRNAs in adipogenesis (Yu et al., 2020a). Scholars can gain more insight into the ways that in cattle, adipose tissue growth and function are impacted through lncRNAs by looking at the epigenetic regulation, functional mechanisms, and molecular mechanisms of lncRNAs in adipogenesis (Li H et al., 2020). With this understanding, methods for influencing adipogenesis and improving beef production qualities in cattle may be developed (Kang et al., 2020). Researchers can pinpoint the lncRNAs responsible for adipocyte differentiation and lipid metabolism by studying their epigenetic regulation, functional roles, and molecular mechanisms in beef cattle’s adipogenesis (Guo et al., 2017; Zhang et al., 2023). This information can be used to create focused interventions targeted at raising the productivity and caliber of beef production, as well as providing insight into the oversight networks and biological processes underpinning the adipogenesis in beef cattle (Nolte et al., 2019). Moreover, therapeutic strategies for lowering fat accumulation and improving the quality of meat in beef cattle may be developed by targeting and altering specific lncRNAs associated with adipogenesis (Zhang et al., 2019b; Ma X et al., 2023). Furthermore, as evidence of similar mechanisms and pathways between adipogenesis in humans and farm animals grows, comprehending the role of lncRNAs in the process of adipogenesis can facilitate the development of tailored approaches to address obesity and related metabolic issues in humans (Wang W et al., 2021; Zhai et al., 2021).

Several investigations have revealed particular lncRNAs that exhibit differential expression in beef cattle during adipogenesis. These long noncoding RNAs have been demonstrated to coordinate the adipogenic pathway through interactions with transcription elements, chromatin modifiers, and other regulatory proteins (Shen et al., 2023; Ma X et al., 2023). Clarifying the intricate regulatory networks that govern the process of adipogenesis in beef cattle requires an understanding of the molecular mechanisms and functional functions of these complex noncoding RNAs (Zhang et al., 2019b; Wang J et al., 2020). The function, molecular mechanisms, and epigenetic regulation of lncRNAs in beef cattle adipogenesis have the potential to fundamentally alter our knowledge of the development of adipose tissue and the effectiveness of beef production (Jiang et al., 2020b; Kang et al., 2020). These epigenetic modifications may impact the accessibility of the genes involved in adipogenesis, which may impact the efficient production of beef in cattle as well as the development of adipose tissue (Zhang et al., 2019b; Jiang et al., 2020b; von Braun et al., 2023). Overall, studies on the lncRNAs and epigenetic factors: their role in regulating the adipocytes in bovine have the potential to advance our knowledge of the development of adipose tissue and increase the productivity of cattle raised for beef production (Cai W et al., 2018; Nolte et al., 2019; Jiang et al., 2020b).

Scholars worldwide now place greater importance on studying the molecular mechanisms underlying the control of adipogenesis due to recent advances in the field of epigenetics. Despite significant progress in our knowledge of the genetic and environmental influences on the adipogenis of beef cattle, little is known about the lncRNAs and epigenetic factors: their role in regulating the adipocytes in bovine. The importance of epigenetic control in adipogenesis in beef cattle has been suggested by certain findings; however, a thorough synthesis and analysis of the most recent research in the field is conspicuously lacking. Thus, the primary goal of this study is to deepen our understanding of how epigenetic factors and lncRNAs regulating the differentiation of cattle adipocytes, shedding light on their potential as targets for genetic improvement and production optimization with the specific goals listed as follows: (a) To articulate the lncRNAs and epigenetic factors in bovine adipocytes (b) to articulate their role in regulating the adipocytes in bovine. (c) To shed light on the regulation mechanism of lncRNAs in the adipogenesis of beef cattle. This review’s information came from a range of secondary sources, including relevant books, scholarly articles, and internet resources. The information was then presented in accordance with the goals of this paper.

## 2 Overview of adipogenesis in beef cattle

Adipogenesis, the process of fat cell development, is a crucial physiological mechanism in beef cattle that directly impacts meat quality and quantity (Wang L et al., 2020). Understanding the intricacies of adipogenesis is essential for optimizing production and ensuring high-quality beef products for consumers (Liu et al., 2020). It involves the complex interplay of various genetic, environmental, and nutritional factors that influence the deposition of adipose tissue and ultimately the marbling and composition of beef cuts (Yu et al., 2019; Raza et al., 2022). Further exploration of the molecular and cellular mechanisms involved in adipogenesis has the potential to offer valuable insights for enhancing beef production efficiency and meeting changing market demands (Park et al., 2018).

The complex process of adipogenesis in beef cattle involves preadipocyte maturation into adipocytes, leading to the accumulation of adipose tissue (Sufianov et al., 2023). This process is influenced by a wide range of variables, including genetics, hormones, and diet. Increasing the quantity and quality of meat that beef cattle produce requires an understanding of the cellular and molecular mechanisms that control adipogenesis. An animal’s propensity for adipogenesis is largely determined by genetic factors. Research has shown that certain breeds of cattle exhibit a higher propensity for marbling, which has a direct bearing on the adipogenic process (Bulls, 2020; Ren et al., 2020a). Moreover, the effectiveness of adipogenesis in beef cattle can be influenced by specific gene expression linked to lipid metabolism and the differentiation of adipocytes (Liu et al., 2020; Wang Y et al., 2021; Kuraz Abebe et al., 2024). Adipogenesis is significantly influenced by environmental and dietary factors in addition to genetic factors (Ladeira et al., 2016). Environmental stressors, such as heat and cold, can affect hormone levels, which in turn influence the deposition of adipose tissue. Similarly, the nutrition provided to beef cattle, including the composition of their diet and feeding strategies can significantly modulate the rate and extent of adipogenesis (Park et al., 2018).

**FIGURE 1:**
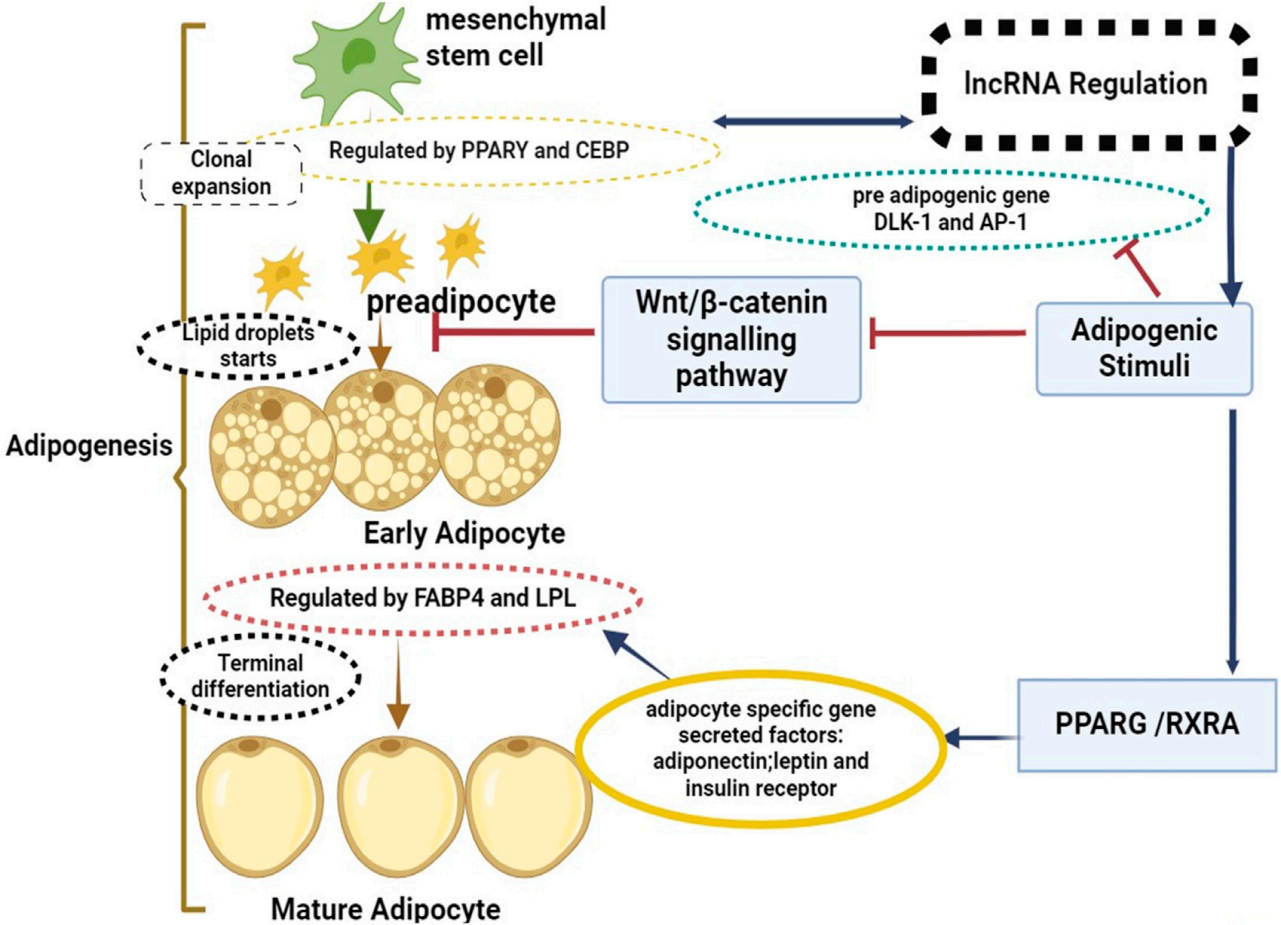
Mesenchymal stem cells to mature adipocytes: a mechanism of lncRNAs regulation and the shift from adipogenesis.

Exploring the molecular signaling pathways and key regulators involved in adipogenesis can offer critical insights into potential targets for genetic selection, as well as opportunities for nutritional and management interventions to optimize adipose tissue deposition (Cai W et al., 2018; Raza et al., 2022). This deeper understanding of adipogenesis will aid in the creation of innovative approaches for enhancing the efficiency of beef production and meeting the increasingly sophisticated demands of consumers for high-quality beef products (Li Y et al., 2020; Wang Y et al., 2021). Figure 1 illustrated that the process of adipogenesis, which involves the differentiation of mesenchymal stem cells into mature adipocytes, is strictly regulated through mechanisms like epigenetic changes, transcriptional regulation, and post-transcriptional regulation, lncRNAs have been revealed to play key roles in this regulation, operating as both positive and negative regulators (Sufianov et al., 2023). Modulation of chromatin structure and epigenetic changes is one way that lncRNAs control the process of adipogenesis (Kornfeld and Brüning, 2014). For instance, lncRNAs can suppress the expression of anti-adipogenic genes or boost the expression of adipogenic genes by interacting with chromatin remodeling complexes. Furthermore, by interacting with transcription factors or RNA polymerase complexes, lncRNAs can function as transcriptional regulators and affect the expression of adipogenic genes (Sun and Lin, 2019). Arrows link these processes to certain areas such as mesenchymal stem cells to pre adipocytes known as “clonal expansion phase” Pluripotent stem cells differentiate into preadipocytes with traits of the adipocyte lineage during clonal expansion. The cell’s capacity to differentiate into different cell types is limited by this transformation. The Wnt/β-catenin signaling pathway regulatory factors, positive negative delta-like one homolog (DLK1) and activator protein-1 (AP-1) are involved in this process. Arrows link from early adipocytes to mature adipocytes known as “An increase in secretory capacity, an increase in insulin sensitivity, and modifications to cell shape are all part of the “terminal differentiation phase”. The cell’s internal fat build-up and transition to a more rounded shape are additional features of this phase. CCAAT/enhancer-binding protein beta and delta activation is the first step towards the expression of key adipogenesis factors, including peroxisome proliferator-activated receptor gamma and CEBP alpha. This initiates a cascade of transcriptional events. These transcription factors also enhance the expression of lipid metabolism-related genes, including fatty acid-binding protein 4 (FABP4) and lipoprotein lipase (LPL).

WNT is the wingless-type Wnt/β-catenin signaling pathway; DLK-1 is the delta-like one homolog; and AP-1 is the activator protein-1. It also comprises lipoprotein lipase (LPL), retinoic X receptor alpha (RXRA), peroxisome proliferator-activated receptor gamma (PPARγ), enhancer-binding protein beta (PPARγ), and fatty acid-binding protein 4 (FABP4).

### 2.1 The identification of genes that regulate the adipocyte differentiation process in cattle

Differentiation of adipocytes is crucial for intramuscular fat deposition in bovine species. Moreover, the CB1, cannabinoid receptor 1; PPARγ2, peroxisome proliferator activated receptor gamma; PLIN1, perilipin 1; HSL, hormone-sensitive lipase (CB1/PPARγ2/PLIN1/HSL) pathway has been implicated in regulating lipid metabolism and fat deposition across various cattle breeds (Baik et al., 2023). These findings suggest that the CB1/PPARγ2/PLIN1/HSL pathway and epigenetic repression are potential mechanisms influencing the accumulation of fat in cattle raised for beef (Liu et al., 2021). To completely understand the molecular regulatory mechanisms governing the accumulation of fat in cattle raised for beef, more investigation is necessary. Studying the genes and markers involved in this procedure is crucial for understanding the deposition of beef fat and for enhancing the quality of the meat (Ren et al., 2020b; Cheng et al., 2023). Potential genes regulating adipocyte differentiation have been found by researchers using high-throughput sequencing and bioinformatics analysis (Jiang et al., 2020a). The peroxisome proliferator-activated receptor is one such gene that is essential forregulating lipid metabolism and adipocyte development. Adipocyte differentiation process Peroxisome proliferator-activated receptor γ, or PPARγ, is a transcription factor that binds to target gene promoter regulatory sequences. It controls preadipocyte differentiation in adipose tissue and is crucial in adipocyte metabolism regulation (Vella et al., 2016). It may be possible to regulate obesity and avert disorders associated with obesity by learning more about the PPARγ structure, expression patterns, and mechanisms of action in adipocyte development. Peroxisome proliferator-activated receptor γ (PPARγ) is the primary regulator of the complicated processes known as adipocyte proliferation and differentiation, which are governed by several genes. As a result of decreased adipose tissue development and functionality, defects in PPARs have been connected to lipodystrophy, obesity, and insulin resistance (Li Z et al., 2022).

Adipocytes differentiation process offer novel approaches to controlling intramuscular fat deposition in beef cattle by shedding light on the molecular mechanisms underlying the adipogenesis of bovine species (Wei et al., 2019; Cheng et al., 2023). Preadipocyte proliferation and differentiation in bovine adipocytes have been found to be regulated by the CDC10 gene. Research suggests that overexpression of CDC10 promotes adipocyte differentiation, while knockdown of CDC10 has the opposite effect (Cheng et al., 2023; Yu et al., 2023). These results imply that improving meat quality in beef cattle by targeting CDC10 may control the deposition of intramuscular fat (Raza, et al., 2022). Proteins and genes such as PPARγ coactivator-1 and PPARγ are examples of those that regulate the maturation of bovine adipocytes (Zhou et al., 2020).

(Table 1). Moreover, adipocyte differentiation and gene expression are controlled by the interplay between transcription factors and sets of co-regulatory proteins that include both activators and repressors (Kang et al., 2020; Qiu et al., 2020). Through investigating alternative splicing, tissue-specific splicing, and the role of HIFs in gene expression and adaptation to hypoxia, researchers may find more genes or markers connected to the differentiation of bovine adipocytes (Mi et al., 2019). Furthermore, multiple isoforms with unique biological roles can result from alternative splicing of genes, like the receptor for advanced glycosylation end products, which further affects adipocyte differentiation (Hu et al., 2023). Furthermore, understanding how specific genes are expressed during synaptic development and neuronal differentiation may help identify the genes and markers that control the differentiation of bovine adipocytes (Huang J et al., 2018) (Table 1).

**TABLE 1:** Function of genes or markers that control the differentiation of bovine adipocytes.

Name of gene	Function/mechanism	Function in adipocytes	References
HOXA9	Downregulated the molecular expression of PPARγ, CEBPα, FABP4, and LPL in addition to the mRNA of these adipogenic markers	Participated in the development and proliferation of pre adipocytes as a negative regulatory factor	He et al. (2024)
FGF10	induced by siFGF10 or overexpressed by Ad-FGF10	detrimental regulator of cattle’s adipogenic process and a putative marker-assisted selection candidate gene	Kaster et al. (2023)
CDC10 (Septin7)	A septin family member implicated in cell division was thought to be a positional and functional candidate gene for beef marbling	promote the growth of cattle’s intramuscular preadipocytes	Cheng et al. (2023)
*KLF6*	It has been specifically demonstrated that during the muscle development and growth of neonates, KLF6 is highly expressed via the TGFB1 (transforming growth factor beta 1) factor signaling pathway	The KLF6 gene significantly increased the expression of genes related to the homeostasis of bovine adipocytes, adipocyte differentiation and regulation, and adipogenesis	Raza et al. (2022)
*ACSL1*	fatty acid (FA) activation, transport, and degradation, as well as lipid synthesis	Controls the synthesis of unsaturated fatty acids (UFA) and the formation of lipid droplets in bovine adipocytes	Bai et al. (2021)
Smad3	Myogenesis and adipogenesis are both inhibited by Smad3. KLF7 and MZF1 likewise function as activators, while KLF6 and KLF15 encourage the differentiation of myoblasts and preadipocytes while inhibiting Smad3 expression and activity	The growth and development of muscle and adipose tissue are negatively regulated by the Smad3 gene	Zhang L et al. (2018)
TORC2	regulated family of proteins that binds to the cAMP response element, important for both adipogenesis and metabolism	Positively regulates the development and differentiation of bovine adipocytes	Khan et al. (2019)
PSMA1	When bovine preadipocytes were differentiating *in vitro* under standard culture conditions, PSMA1 mRNA was expressed differently	Lipid accumulation in adipocytes and preadipocyte differentiation	(S. Li et al., 2019)
TP53INP2	The regulation of adipocyte differentiation by TP53INP2 was additionally facilitated by peroxisome proliferator-activated receptor gamma (PPARγ)	can stimulate autophagy in bovine adipocytes during the early stage of differentiation and can positively control adipocyte differentiation by influencing autophagy	Zhang et al. (2019)

## 3 Epigenetic regulation of beef cattle's fat deposition

The intricate and multifaceted process of manipulating epigenetics to control fat deposition in beef cattle involves the interaction of numerous genetic and environmental factors (Venkatesh et al., 2023). Epigenetic mechanisms such as DNA methylation, histone modifications, and non-coding RNA control play a major role in controlling the expression of genes associated with fat metabolism and deposition (Baik et al., 2023). Enhancing meat quality, production efficiency, and overall profitability for the beef industry can be significantly impacted by understanding of the epigenetic controls over the accumulation of fat in cattle (Wang and Ibeagha-Awemu, 2021; Venkatesh et al., 2023).

Through investigating at how beef cattle’s fat deposition is regulated epigenetically, scientists hope to decipher the underlying molecular mechanisms, pinpoint important regulatory genes and pathways, and devise focused approaches for modifying fat deposition to augment desirable characteristics like marbling and meat tenderness (Corvo et al., 2020; Ren et al., 2020a; Zhao et al., 2020). According to current research, changes in epigenetic mechanisms may have an effect on preadipocyte maturation and release, as well as the storage and release of fat in adipose tissue (Wei et al., 2017; Wang and Ibeagha-Awemu, 2021). Enhancing meat quality, increasing productivity, and ultimately maximizing profits in the beef industry are all significantly impacted by the epigenetic control of adipose tissue deposition in beef cattle (Wang and Ibeagha-Awemu, 2021; Venkatesh et al., 2023). To investigate this further, research studies can focus on profiling the epigenetic landscape of adipose tissue in beef cattle with divergent fat deposition phenotypes (Liu et al., 2020). This might entail profiling histone modifications, analyzing the expression of non-coding RNAs in adipose tissue samples, and performing genome-wide DNA methylation analysis (Baker et al., 2023). The identification of epigenetic markers linked to characteristics related to fat deposition can provide researchers with important insights into the regulatory mechanisms controlling this intricate biological process (Zhao et al., 2023).

Furthermore, examining how environmental factors, like stress and diet, affect the epigenetic regulation of fat deposition can offer a more thorough comprehension of how genetics and environment interact to shape the phenotypic variations seen in cattle raised for beef (Corvo et al., 2020). As the development of focused epigenetic-based techniques for enhancing the effectiveness of fat deposition and, eventually, the caliber of beef production can result from this integrative approach (Massa et al., 2021). Gene regulation in muscle cells has been associated with H3K27me3, a repressive histone modification that influences mineral levels and is connected to meat quality (Feng et al., 2022). Histone modification and mineral concentration in beef samples have been linked by computational research, which also identified possible regulatory genes impacted by suppressive epigenetic mechanisms (Afonso et al., 2023).

### 3.1 Methylation of DNA related to fat accumulation in beef cattle

One important epigenetic change is DNA methylation that affects a variety of biological processes, including fat accumulation (Zhao et al., 2020). Exploring a link between DNA methylation and the accumulation of fat in beef cattle offers valuable insight into the intricate molecular processes related to this characteristic (Park et al., 2018; Zhao et al., 2020). Studies have demonstrated the association between patterns of DNA methylation in adipose tissue and important genes involved in fat metabolism and storage (Wang and Ibeagha-Awemu, 2021). Moreover, examining how DNA methylation may impact diverse physiological processes in beef cattle, beyond just fat accumulation, can reveal its broader significance for animal health and production (Huang et al., 2014; Zhao et al., 2020). Furthermore, research into the possible use of DNA methylation as a biomarker for the accumulation of fat in beef cattle can be useful for genetic selection plans and breeding initiatives (Zhao et al., 2020).

The utilization of DNA methylation as a marker for fat accumulation in beef cattle has the potential to significantly transform breeding programs and genetic selection approaches (Raza et al., 2019). By pinpointing and selecting animals with favorable DNA methylation patterns linked to efficient fat deposition, breeders can enhance the overall quality and profitability of their herds (Baik et al., 2017). This could result in increased meat production, improved beef quality, meeting consumer needs, and boosting industry earnings (Wang and Ibeagha-Awemu, 2021). Additionally, understanding how DNA methylation affects fat deposition may have health effects on human beings (Fan et al., 2020). Additional research investigating the conservation and similarities in DNA methylation patterns between beef cattle and humans could offer valuable insights into obesity-related conditions and possibly contribute to developing new strategies for managing and preventing obesity in humans (Zhao et al., 2015; Raza et al., 2019). While much research has been done on the connection between DNA methylation and beef tenderness, there still remains much to be elucidated about this aspect. Additional research into the relationship between DNA methylation and tenderness in beef may yield valuable information about the underlying molecular processes associated with this important quality characteristic (Huang et al., 2014; Zhao et al., 2020).

Variations in prenatal nutrition and genetic predisposition for residual feed intake have also been found to modify DNA methylation patterns in postnatal tissues and sexes; The majority of these alterations affect genes linked to the IGF2 receptor and insulin-like growth factor 2 (IGF2R) (Halušková et al., 2021). Weight and adiposity have been closely linked to IGF2R expression; methylation of the IGF-II gene is correlated with birth weight; and IGF2R expression *in utero* stimulates adipogenesis and fat accumulation during pregnancy (Holly and Perks, 2012). Furthermore, the amount of IGF2R in the bloodstream during childhood has been strongly correlated with fat mass (35), and the methylation status of the IGF2R gene at birth has been associated to early childhood weight. In addition to potentially serving as a protective factor in controlling body fat composition, IGF2R may function as a physiological regulator of preadipocyte development and metabolism. Interestingly, it has been found that IGF-II regulates muscle mass and skeletal muscle cell development in addition to its effects on fat (Wilson and Rotwein, 2006). It could also restrain metabolic risk by favoring muscle formation over that of fat. These findings suggest that DNA methylation may play a part in fat deposition and other traits that are significant to the beef cattle industry (D. Wang et al., 2022).

### 3.2 Histone modifications and chromatin remodeling during adipogenesis

**FIGURE 2:**
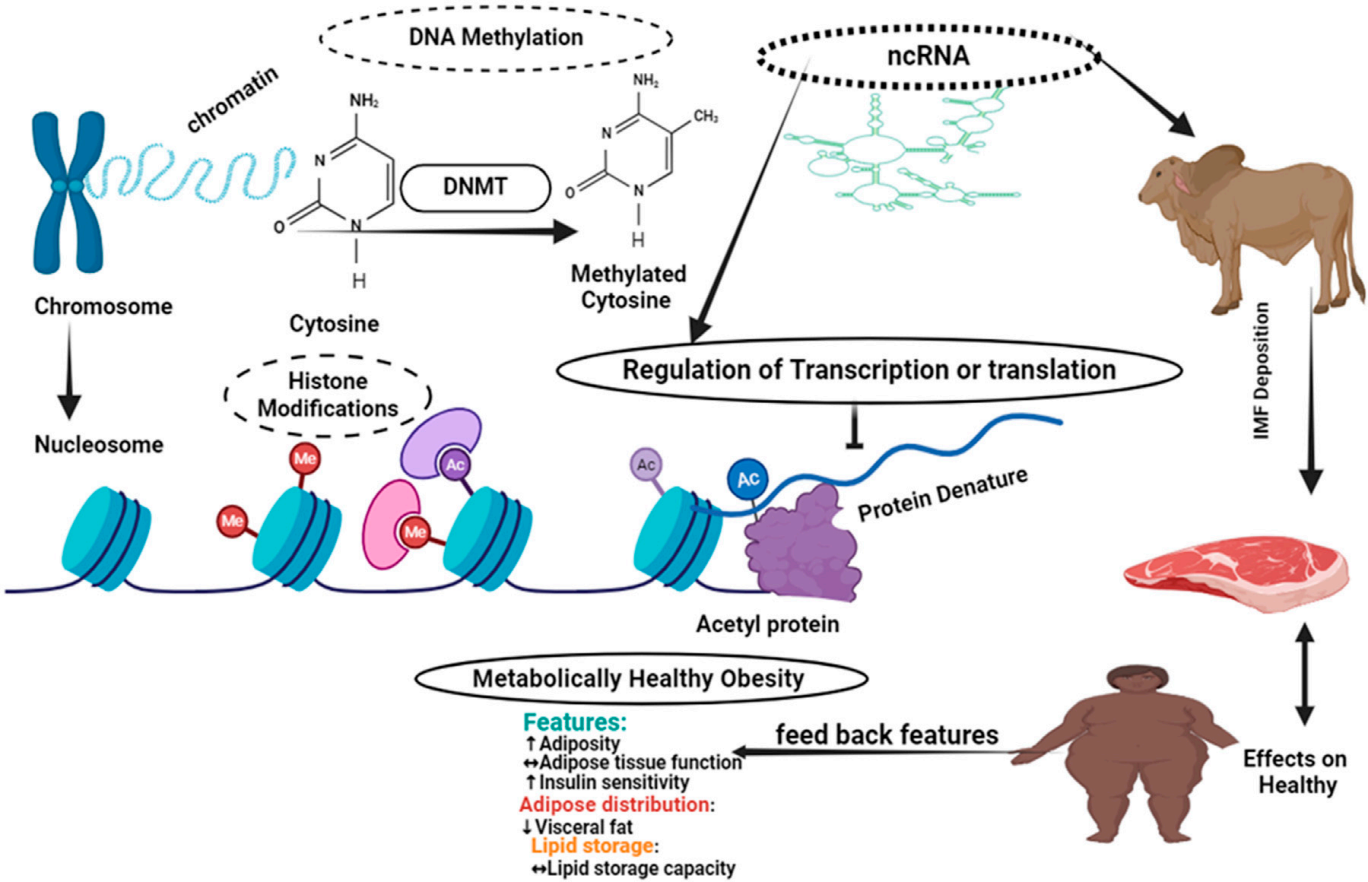
Epigenetic mechanisms influencing intramuscular fat deposition in cattle.

As Figure 2 shows Epigenetic changes are variations in gene expression that are not brought about by changes in DNA sequences. The primary epigenetic mechanisms that alter patterns of gene expression are DNA methylation and histone modifications, including acetylation and methylation. The chromatin architecture, DNA-protein interaction, and DNA-non-coding RNA interaction are all altered as a result of these changes. By regulating gene expression, which in turn influences the accumulation of intramuscular fat, epigenetic mechanisms have an effect on human health (Figure 2).

During adipogenesis, chromatin remodeling and histone modifications are crucial for controlling gene expression (Lee et al., 2023). A key modification involved is histone acetylation, which is linked to open chromatin and active gene transcription (Ong et al., 2020; Wang L et al., 2021). Several investigations have demonstrated that at specific gene locations, histone acetylation is essential for the activation of adipogenic transcription factors like C/EBPα and PPARγ (Huang Q et al., 2018). These factors bind to the promoter regions of genes involved in adipogenesis, promoting their expression and causing preadipocytes to develop into adipocytes (Wang L et al., 2020). Other histone modifications like methylation, phosphorylation, and ubiquitination also contribute to regulating adipogenesis by modifying the structure of chromatin and affecting transcriptional regulator recruitment (Bannister and Kouzarides, 2011; Ibeagha-Awemu and Zhao, 2015). Adipogenesis is significantly influenced by chromatin remodeling complexes like ISWI and SWI/SNF by altering DNA accessibility for regulatory proteins (Ong et al., 2020). They use ATP hydrolysis to reposition nucleosomes and modulate chromatin structure, thus controlling the way adipogenic genes are expressed (Dahlin et al., 2015; Halušková et al., 2021).

To fully understand the molecular mechanisms underlying adipogenesis, one must have a thorough understanding of chromatin remodeling, histone modifications, and adipogenic gene expression. By studying adipocyte development in greater detail, this field of study could yield novel therapeutic approaches for treating obesity and metabolic diseases (Ong et al., 2020). Studying histone modifications and chromatin remodeling in adipogenesis helps us understand the molecular mechanisms that control adipocyte development and gene expression (Possomato-Vieira et al., 2017). This knowledge is essential for developing targeted treatments for metabolic disorders and obesity (Okuno et al., 2013; Ong et al., 2020). Further investigation is required to comprehend the exact mechanisms underlying chromatin remodeling and histone modifications, especially in the adipose tissue of beef cattle.

### 3.3 Cross-talk among epigenetic alterations in adipogenesis in beef cattle

The process of fat cell development in beef cattle is intricate, with various interconnected epigenetic changes (Zhao et al., 2023). Epigenetic modifications in beef cattle’s adipose tissue are crucial for regulating gene expression and influencing beef quality. Several investigations have shown that adipose tissue-derived stem cells exhibit pluripotency-related characteristics and genetic properties that are comparable to those of embryonic stem cells. This implies that there may be room for regeneration and cell type differentiation, including into adipocytes. The creation of adipocytes and the control of lipid metabolism in adipose tissue are fundamental processes facilitated by the peroxisome proliferator-activated receptor (Jandial, 2014; Liu et al., 2020; Devos et al., 2021).

One important modification that affects how genes linked to fat accumulation are expressed is DNA methylation (Zhao et al., 2020). Epigenetic regulation through DNA methylation involves the enzymatic addition of methyl groups to cytosine residues within CpG dinucleotides. This process, catalyzed by DNA methyltransferase enzymes, serves as a regulatory mechanism that can modulate gene expression patterns (Bommarito and Fry, 2019). Methylation in certain regions of a gene can block the binding of transcription factors and other regulatory proteins, thus preventing gene expression; for example, when the promoter region of TP53 is methylated, it inhibits activation by necessary transcription factors. This reduction or silencing of TP53 expression may lead to uncontrolled cell growth and cancer development (Bénard et al., 2003; He et al., 2020). DNA methylation has the potential to impact gene expression associated with fat storage through its influence on the regulation of adipogenic transcription factors. One such example is the peroxisome proliferator-activated receptor gamma, a crucial transcription factor in adipocyte differentiation and lipid metabolism (Houseknecht et al., 2002). DNA methylation can control PPARγ expression and that of other genes related to fat accumulation, primarily by inhibiting the binding of transcription factors when methylated at their promoter regions, hindering gene activation (Loh et al., 2019). Consequently, decreased or silenced PPARγ expression results in impaired adipocyte differentiation and lipid metabolism, potentially contributing to an elevated risk of obesity due to increased fat accumulation. Furthermore, DNA methylation may also affect genes encoding additional adipogenic transcription factors like C/EBPα; this alteration could disrupt both adipocyte differentiation and lipid metabolism processes - further influencing fat buildup (Kim et al., 2015; McAllan et al., 2023).

Histone modifications like acetylation and methylation have an impact on the regulation of gene expression related to fat formation (Chen X et al., 2021). Recent research in beef cattle has shown the complex interplay of multiple epigenetic modifications during adipogenesis (Cai W et al., 2018; Ren et al., 2020a). Epigenetic changes control gene expression and cellular specialization in adipogenesis through DNA methylation, histone modifications, chromatin remodeling, and microRNA activity. DNA methylation involves adding a methyl group to cytosine residues at CpG dinucleotides, often leading to gene suppression (Moore et al., 2013). Histone modifications like acetylation, methylation, and phosphorylation regulate the expression of adipogenic genes by affecting transcriptional activation or repression (Delage and Dashwood, 2008). Chromatin remodeling also emerges as a key player in adipogenesis, influencing the accessibility of DNA to transcription factors and other regulatory proteins. The dynamic reorganization of chromatin structure allows for the activation or repression of specific genes essential for adipocyte development and function (Siersbæk et al., 2012). Furthermore, emerging evidence suggests that microRNAs (miRNAs) play a significant role in post-transcriptional regulation during adipogenesis. These small non-coding RNAs can fine-tune gene expression by targeting mRNAs for degradation or by inhibiting their translation, thereby sculpting the adipogenic transcriptome (Maurizi et al., 2018; Wang et al., 2020). Understanding the complex interaction of these epigenetic processes provides valuable understanding of the precise control of adipogenesis and suggests potential treatments for obesity and related metabolic conditions (Kasinska et al., 2016; Rosen et al., 2018; Pant et al., 2021).

Furthermore, environmental factors like diet and stress can impact epigenetic alterations, affecting adipogenesis in beef cattle. Diet can impact epigenetic changes by consuming specific nutrients or bioactive substances. For example, research has indicated that a lack of folate may result in reduced DNA methylation, disrupting normal gene expression and potentially playing a role in conditions like cancer (Kandi and Vadakedath, 2015; Flavahan et al., 2017). Additionally, chronic stress can cause alterations to DNA methylation patterns, particularly affecting genes related to the regulation of the stress response. These modifications may have lasting impacts on gene expression and could potentially contribute to the onset of anxiety and depression due to stress-related disorders (Li et al., 2019; Park et al., 2019). Several studies have demonstrated that specific processes play a role in how diet and stress affect epigenetic changes. One way in which diet impacts epigenetic changes is by altering histone modifications. For instance, certain natural compounds present in fruits and vegetables, such as polyphenols, have been found to influence histone acetylation or methylation patterns, leading to potential effects on gene expression (Arora et al., 2020; Sęczyk et al., 2021; Jia et al., 2022). Another way through which diet affects epigenetic changes is by regulating microRNAs.

Investigating the interplay among histone modifications, DNA methylation, and non-coding RNAs in reaction to environmental cues yields valuable insights into the formation of adipose tissue in beef cattle (Cai H et al., 2018). This understanding can help develop targeted strategies for enhancing beef production and quality (Khan et al., 2020). Non-coding RNAs, especially microRNAs, have significant regulatory roles in adipogenesis in beef cattle. These tiny RNA molecules have the ability to impact the expression of important genes involved in the formation of fat cells (Zhu et al., 2021). Knowing how different epigenetic modifications interact is necessary to improve the quality of beef and develop efficient management strategies for cattle breeding. Understanding the interactions of epigenetics in enhancing beef quality has the potential to transform management approaches in the beef industry. Epigenetics is essential for regulating gene expression and can greatly influence characteristics related to beef quality, including tenderness, marbling, and flavor (Wang and Ibeagha-Awemu, 2021). By further investigating the mechanisms of epigenetics involved, researchers and professionals in the industry can acquire valuable knowledge on how to enhance beef production and refine management techniques (de Souza et al., 2022). A better understanding of epigenetic interactions can help solve long-standing issues in improving beef quality. For instance, by identifying specific epigenetic markers associated with desirable traits, breeders and geneticists can develop targeted selection methods to enhance beef quality in livestock (Ibeagha-Awemu and Zhao, 2015; Liu et al., 2015). This knowledge also has the potential to influence the development of new feed formulas and farming practices that promote favorable epigenetic changes in beef cattle, leading to improved meat quality (Liu et al., 2015; Murdoch et al., 2016; Triantaphyllopoulos et al., 2016). Understanding epigenetic interactions has wide-ranging implications that go beyond improving beef quality. It also impacts animal welfare and environmental sustainability in the beef industry.

## 4 lncRNAs regulation mode

lncRNAs possess the crucial role of regulating gene expression through a range of complex processes, such as interactions with proteins, RNA, and DNA (Statello, C.-J. Guo, et al., 2021). They can act as scaffolds to form ribonucleoprotein complexes, modulating chromatin structure and gene expression. Furthermore, lncRNAs work as sequestering microRNAs, influencing mRNA regulation, and ceRNAs (Qin et al., 2020). ceRNAs compete for miRNA binding, influencing target gene expression. This interplay is important for various biological processes including development, differentiation, cell proliferation, metabolism, and disease. ceRNAs can act as “sponges” for miRNA molecules by capturing and obstructing their interaction with target mRNAs, resulting in increased expression of these genes (Li et al., 2015; An et al., 2017). They also regulate the levels of gene expression at the transcriptional and post-transcriptional levels by a number of different mechanisms, including mRNA decay and histone modification (Marchese et al., 2017).

The field of molecular biology is increasingly focusing on lncRNAs because of their important roles in gene regulation (Zhang et al., 2019). Extended beyond 200 nucleotides and devoid of protein coding, these RNA molecules are essential for various cellular functions, including transcriptional regulation, post-transcriptional modulation, and chromatin remodeling (Ransohoff et al., 2016). Through their interactions with chromatin-modifying complexes, long non-coding RNAs contribute significantly to chromatin remodeling, which can alter the epigenetic landscape of the genome and impacts patterns of gene expression (Zeng et al., 2018). This emphasizes the significance of long non-coding RNAs in shaping cellular transcriptional profiles through their regulatory role in controlling chromatin structure and accessibility (Qin et al., 2020).

In addition, lncRNAs have also been linked to the control of transcriptional processes. They can serve as frameworks that bring together transcription factors, RNA polymerase, and other regulatory proteins, thus aiding in the assembly of transcriptional complexes at particular genomic sites (Statello et al., 2021). Furthermore, long non-coding RNAs can adjust the activity of transcription factors through direct connections, thereby influencing gene expression with precision (Marchese et al., 2017).

Further evidence suggests that lncRNAs influence mRNA stability, splicing, and translation, all of which play a role in the control of post-transcription (Long et al., 2017). Certain lncRNAs have the ability to attach to microRNAs and prevent them from inhibiting their target mRNAs, thereby acting as ceRNAs. This complex network of interactions highlights the diverse functions of lncRNAs in shaping the cellular transcriptome (Schmitz et al., 2016). In conclusion, lncRNAs have diverse molecular mechanisms of action, including chromatin remodeling, transcriptional regulation, and post-transcriptional modulation. Understanding their function provides valuable insights into gene regulatory networks’ complexity and potential for therapeutic interventions in various diseases.

### 4.1 Regulation of epigenetic modification level

The control of epigenetic modifications is a complex process crucial for gene expression and cellular identity (Venkatesh and Makky, 2020). Epigenetic alterations are crucial for controlling gene activation and preserving cell characteristics. A fundamental aspect of modulating epigenetic modification levels involves the dynamic interaction among creators, interpreters, and eliminators of these modifications (Fardi et al., 2018). Creators, like DNA methyltransferases and histone methyltransferases, apply epigenetic tags to DNA and histone proteins. Interpreters, such as chromatin remodeling complexes and transcription factors, decode these tags to initiate or suppress gene activity (Allis and Jenuwein, 2016; Jambhekar et al., 2019).

Eliminators, like DNA demethylases and histone deacetylases, erase these tags to reset the epigenetic pattern. These key concepts and mechanisms are crucial for unraveling the complex regulation of epigenetic modification levels and their implications for various biological processes and diseases (Kim and Kaang, 2017; Youn, 2017). DNA methylation and histone acetylation are regulated by mechanisms ensuring their proper functioning in the cell (Xiong et al., 2020). These mechanisms include enzymatic activities that add or remove epigenetic marks, as well as the recruitment of these enzymes to specific genomic loci (Friese et al., 2019). Enzymes play a key role in adding or removing epigenetic marks, such as DNA methyltransferases adding methyl groups to specific regions of the DNA, and demethylases removing these marks (Friese et al., 2019). Similar to this, histone deacetylases and acetyltransferases control the acetylation of histone proteins, affecting chromatin structure and gene accessibility (Pennings et al., 2019). Additionally, the degree of epigenetic modifications can be influenced by the connection between additional regulatory proteins and transcription factors to particular DNA sequences (Bemer, 2018). Furthermore, non-coding RNAs, chromatin remodeling complexes, and signaling pathways contribute to the regulation of epigenetic modifications (Allis and Jenuwein, 2016). These regulatory networks enable cells to adjust the epigenetic landscape in response to internal and external cues, maintaining cellular homeostasis and adapting to environmental changes (Moosavi and Motevalizadeh Ardekani, 2016).

### 4.2 Transcriptional regulation

Transcriptional regulation is the control of gene expression at the transcriptional level. lncRNAs are crucial for controlling gene expression in a range of biological functions within this regulatory network (Long et al., 2024). Several lncRNAs have been found to participate in beef cattle adipocytes, influencing gene expression (Alexandre et al., 2020). One example is lncRNA-ADINR, which is upregulated during adipogenesis in beef cattle adipocytes and has been observed to facilitate adipocyte differentiation by interacting with let-7 microRNA (Jiang et al., 2020a). Another lncRNA involved in bovine adipogenesis is lncRNA-SRA, acting as a coactivator of PPARγ and enhancing its transcriptional activity. Additionally, lncRNA-HOTAIR has been linked to promoting adipocyte differentiation through modulation of miR-1277-5p-regulated genes COL5A1 and CXCR4 that influence immune cell infiltration levels (Squillaro et al., 2020; Lu et al., 2021). Furthermore, research has identified LncRNA-MALAT1 as a regulatory factor impacting beef cattle adipogenesis by sponging miR-125a and consequently upregulating IL-21R/JAK2/STAT3 signaling associated with enhanced fat cell differentiation (Muniz et al., 2022a). lncRNAs impact transcriptional regulation by serving as scaffolds for protein complexes involved in chromatin remodeling and transcriptional activation or repression. lncRNAs can affect DNA accessibility to the transcriptional machinery and modulate gene expression through interactions with regulatory proteins, chromatin-modifying enzymes, and transcription factors (Chen et al., 2019).

Additionally, lncRNAs can function as recruiters of chromatin-modifying complexes to particular genomic loci. Epigenetic modifications such as histone acetylation or methylation and DNA methylation are so established. These have a significant effect on the transcriptional landscape of the cell, affecting how cells develop and react to external stimuli (Rinn, 2014; Marchese et al., 2017). Moreover, through modifying their interactions and altering the spatial arrangement of the genome, lncRNAs regulate transcriptional enhancers and promoters (Long et al., 2017). Through involvement in long-range chromatin interactions, long non coding RNAs can help create active or repressive chromatin domains, effectively controlling gene expression patterns (Ransohoff et al., 2016). The intricate roles of lncRNAs in transcriptional regulation emphasize their importance in shaping gene expression patterns and warrant further investigation into their mechanisms and potential applications (Kornfeld and Brüning, 2014).

### 4.3 Post-transcriptional regulation

lncRNAs have a significant impact on gene expression regulation at the post-transcriptional level and are the subject of extensive research due to their diverse roles in cellular processes (Statello et al., 2021). These functions include involvement in mRNA splicing, transport, localization, stability, and translation. Furthermore, long non coding RNAs can influence microRNA activity as well as act as ceRNAs, ultimately affecting protein-coding gene expression (Heidari-Ezzati et al., 2024). Specific lncRNAs interact with various RNA-binding proteins to carry out these functions. For instance, XIST is crucial for X chromosome inactivation by recruiting the Polycomb Repressive Complex two to silence gene expression on one of the X chromosomes in females. Similarly, MALAT1 interacts with multiple RNA-binding proteins such as hnRNP C and serine/arginine-rich splicing factor 1 to regulate alternative splicing and promote nuclear speckle formation. NEAT1 plays a pivotal role in paraspeckle formation by interacting with paraspeckle assembly proteins and serving as a scaffold for their recruitment to nuclear paraspeckles” (Hirose et al., 2013; Pisani and Baron, 2019; 2020).

RNA-binding proteins interact with lncRNAs to control the splicing of mRNA, translation, and stability, which is a critical aspect of post-transcriptional regulation (He et al., 2021). This regulation impacts various biological pathways and cellular functions, adding complexity to the understanding of gene expression and emphasizing the significance of researching these non-coding RNA molecules for understanding biological functions and their dysregulation in diseases (Lu et al., 2021). Moreover contributing to their role in post-transcriptional regulation, long non-coding RNAs also regulate alternative splicing, RNA editing, and miRNA-mediated gene silencing (Zhang et al., 2019b). The role of lncRNAs in modulating the relationship between different levels of gene regulation, such as transcriptional control and epigenetic modification, has been highlighted in recent studies. This variety of engagement highlights the significance of lncRNAs as adaptable regulators of gene expression (Guo, et al., 2021).

The interplay of RNA and microRNA is important for regulating cellular processes, including lipid metabolism (Zhang and Wu, 2020). Research has demonstrated that miRNAs can regulate the expression of genes related to adipocytes’ production, absorption, and storage of lipids (Yu et al., 2020b). This interaction influences the capacity of bovine lipocytes to store lipids and may contribute to metabolic irregularities associated with obesity-related diseases in cattle (Kandhro et al., 2017). Understanding the specific mechanisms and implications of RNA-microRNA interaction in bovine lipocytes is crucial for developing therapeutic approaches to modulate lipid metabolism and address obesity within cattle populations (Guo et al., 2021). Adipocyte differentiation, metabolic integration, insulin resistance, and appetite regulation are all significantly influenced by miRNAs.

This could advance our knowledge of bovine lipocytes and aid in the creation of focused treatment plans for metabolic disorders in cattle linked to obesity. Recent research has demonstrated that specific miRNAs, such as miR-27a and miR-143, can directly target genes that bovine lipocytes use for lipid metabolism (Zhang L et al., 2020).

These miRNAs directly target genes that are engaged in lipid metabolism, impacting the pace of fat buildup and differentiation in adipocytes. For example, research demonstrates that miR-27a influences genes such as peroxisome proliferator-activated receptor gamma, fatty acid synthase, and adipose tissue triglyceride lipase (Lin et al., 2013; Zhu et al., 2014). These genes play a significant role in lipid metabolism regulation, with peroxisome proliferator-activated receptor gamma implicated in the differentiation of adipocytes and synthesis of fatty acids, fatty acid synthase responsible for producing fatty acids, and adipose tissue triglyceride lipase involved in breaking down triglycerides into free fatty acids (Kawai et al., 2010). On the other hand, miR-143 targets genes like acyl-CoA synthetase long-chain family member one and acetyl-CoA carboxylase alpha which are related to fatty acid uptake and synthesis. By targeting these genes, miR-27a and miR-143 can influence the expression and activity of crucial enzymes and transcription factors associated with lipid metabolism, thereby impacting fat storage rate and utilization in bovine lipocytes (Wang et al., 2011; Zhang M et al., 2018; Li et al., 2022).

These specific miRNAs cause a decrease in fat accumulation in bovine lipocytes by suppressing the expression of genes involved in adipogenesis and lipid uptake (Khan et al., 2020). Dysregulation of certain miRNAs has been identified in obese cattle, suggesting their potential involvement in obesity-related metabolic disorders within bovine lipocytes (Fei et al., 2023).

By comprehending the intricate mechanisms of RNA-miRNA interactions, researchers can pinpoint specific regulatory pathways that influence fat metabolism. This understanding can then be applied to develop precise breeding strategies, targeting particular genes or miRNAs for manipulation to improve desired fat metabolism in cattle. For instance, breeders may selectively mate cattle with genetic variations that promote more efficient fat metabolism, resulting in enhanced body composition and reduced risk of obesity and related ailments (Mir et al., 2020; Ren et al., 2020b). Furthermore, this study has the potential to contribute to the creation of precise nutritional strategies for cattle. Through the identification of crucial miRNAs associated with fat metabolism, scientists can create dietary interventions that control the activity of these miRNAs in order to enhance healthy fat storage and metabolic processes in cattle. These targeted nutritional approaches may involve developing specialized feed formulations or incorporating specific dietary elements that interact with the identified miRNAs to oversee fat metabolism (Yang et al., 2018; Wang L et al., 2020).

Additionally, recent research has demonstrated the potential for manipulating miRNA expression as a therapeutic approach for improving metabolic health in bovine lipocytes (Huang Y et al., 2022). For instance, targeted delivery of anti-miRNAs or synthetic miRNA mimics could be utilized to modulate the expression of specific miRNAs and regulate lipid metabolism in bovine lipocytes (Yang et al., 2017). These interventions may hold promise in mitigating obesity-related metabolic disorders and improving overall health outcomes in cattle. Understanding the mechanisms and implications of RNA-microRNA interaction is crucial for developing therapeutic approaches to modulate lipid metabolism and address obesity within cattle populations (Guo et al., 2021; Chen X et al., 2021; Fei et al., 2023).

## 5 Long non-coding RNAS in bovine adipocytes

lncRNAs are essential regulators of gene expression that affect a variety of biological processes, including adipogenesis (Li H et al., 2020; Zhang W et al., 2023). Optimizing the efficiency of beef production requires an understanding of the molecular mechanisms driving adipogenesis, particularly the role of lncRNAs (Li H et al., 2020; Hu et al., 2023).

lncRNAs are now known to be important regulators in the intricate process of adipogenesis, which involves the formation of fat cells (Table 2). Recent studies have revealed the various functions that lncRNAs fulfill in controlling adipocyte differentiation and lipid metabolism. Unraveling the detailed mechanisms through which lncRNAs impact adipogenesis is essential for progressing our understanding of beef production efficiency (Jiang et al., 2020b; Wang L et al., 2021). Beef production relies heavily on the buildup of fat in muscles, which plays a substantial role in determining meat quality and customer satisfaction. Studying the link between lncRNAs and adipogenesis can provide valuable insights for enhancing efficiency in beef production. Through investigating the intricate regulatory interactions involving lncRNAs and their impact on adipocyte growth, researchers and industry practitioners might enhance both the quantity and quality of beef products (Nolte et al., 2019). Further investigation into the particular long non-coding RNAs implicated in adipogenesis and their subsequent targets could provide new prospects for manipulating fat accumulation in beef cattle (Ma et al., 2023). This increased comprehension might eventually result in the creation of original approaches to enhance feed efficiency, decrease production expenses, and fulfill consumer expectations for premium beef quality (Li Y et al., 2022).

**TABLE 2:** Current studies conducted on long intergenic non-coding RNAs; lncRNAs since 2020, including novel lncRNAs.

Study	lncRNA	Model animal	Number of analyzed sample	Methods	Number of lncRNA/Gene detected	Features/categories	Cells/tissue	Function	Key finding
Asselstine et al. (2024)		Holstein dairy cows	12	RNA-seq analyses	94		milk somatic cell	Regulating milk somatic cells	Identifying lncRNA regulatory components that may play a functional role in the immune system’s reaction to mastitis in dairy cows of the Holstein breed
Ma X et al. (2023)	(BIANCR)	Qinchuan Cattle	12	RNA-seq analyses	7,035	Distributed uniformly throughout the cytoplasm and nucleus	Bovine Heart, liver, spleen, lung, kidney, subcutaneous fat, and longissimus dorsi muscle (LDM)	Knockdown of intramuscular adipogenesis is achieved through the regulation of the ERK1/2 signaling pathway	Novel lncRNA and BIANCR were identified as important Regulatory function in Adipogenesis of bovine intramuscular adipocytes
Zhang et al. (2023)	LNCBNIP3	Qinchuan and japanes black cattle	6	RNA-seq analyses	660	Located in the cytoplasm and nucleus	spleen, liver, lung, kidney, heart, subcutaneous fat, longissimus muscle, and intramuscular fat	Inhibited Bovine Intramuscular Preadipocyte Proliferation	In the intramuscular fat of Qinchuan and Japanese black cattle, lncBNIP3 is expressed differently. It inhibits bovine intramuscular preadipocyte proliferation by regulating the cell cycle, DNA replication pathways, and CDC6 expression
Ma X et al. (2023)	LNC000100	Angus cattle	6	transcriptome analysis	37,115	Located by RNA-FISH within the nucleus	longissimus dorsi muscle	inhibited cell proliferation and promoted cell differentiation	Longitudinal dorsal muscle in fetuses is downregulated by lncRNA, which is abundantly expressed in muscle tissue and greatly stimulates myoblast proliferation and differentiation
He et al. (2023)	MSTRG.8190.1and XR_001351700.1	white yaks	18	RNA-seq analyses	1925	Located in the cytoplasm and nucleus	longissimus dorsi muscle tissue	Control IMF deposition and muscle growth	Identify the mRNAs and LncRNAs that are involved in the formation and development of the longissimus dorsi muscle tissue in Tianzhu white yaks at different ages
Chai et al. (2023)	G8110	Bama pigs	48	RNA-seq analyses	2,730	Situated close to NFE2L1’s upstream regulatory area	bonestem cells from the hind legs	adipogenic differentiation through the enhancement of NFE2L1 regulation	LncRNA G8110 regulates the expression of the NFE2L1 gene positively, which affects lipid metabolism, immunological responses, and fat storage in mesenteric adipose tissue
Tang et al. (2023)	MSTRG.310246.1	goat	6	RNA-seq analyses	2,936	Located in both the nuclear and cytoplasmic compartment	perirenal adipose tissues	enhanced thermogenesis and differentiation in brown fat cells	Compared to white adipocytes, brown adipocytes in goats that were treated with MSTRG.310246.1 had increased differentiation and thermogenic activity
Huang et al. (2023)	ACART	Mice	5	RNA-seq analyses	3,192	mainly located in cytoplasm	subcutaneous, perirenal, and epididymal fat	Reduced PPARγ and C/EBPα expression and inhibited preadipocyte development	The downregulation of Acart expression partially inhibits the proliferation and differentiation of 3T3-L1 preadipocytes
(J. Zhang et al., 2023)	LNCA2B1	cattle	6	RNA-seq analyses	301	located primarily in the cytoplasm and in the nucleus	bovine skeletal muscle satellite cells	regulates myoblast differentiation	This work discovered a novel long noncoding RNA (lncRNA) called lncA2B1, which interacts with the common binding proteins HNRNPA2B1 to regulate myogenesis in conjunction with miR-206
Feng et al. (2023)		Laiwu pigs	3	deep sequencing	1,258		intramuscular and subcutaneous adipose tissue	regulate adipocyte differentiation and metabolism	1,258 lncRNAs with differential expression were found in subcutaneous and intramuscular fat; 666 of these were downregulated and 592 of them were upregulated
Varela-martínez et al. (2023)		Goat	7	RNA-seq analyses	5,892	Evenly distributed across chromosomes	Brain tissue	Initiates brain development	lncRNAs were expressed in brain tissues, resulting in the identification of 794 known lncRNAs and 5,098 new potential lncRNA sequences
Wu et al. (2023)		Cashmere Goats	12	RNA-seq analyses	129		skin tissue	hair follicle growth and development	In skin tissue samples from Ziwuling black goats and Liaoning cashmere goats, 129 lncRNAs were expressed differently
Wang D et al. (2023)		Bama Pig	23	RNA-seq analyses	82	Located on chromosome	Spleen tissue	regulate spleen development, such as myofibril assembly and sarcomere	82 differently expressed long non-coding RNAs and 142 differentially expressed genes in total were found. It has been established that the target gene ACTN2 is an essential candidate gene for promoting spleen expansion in ZBED6 knockout pigs
Dehghanian Reyhan et al. (2023)		Shandong black cattle and Luxi cattle	6	RNA-seq analyses	43	Located on chromosome	longissimus dorsi muscle	regulate molecular mechanisms associated with intramuscular fat	By integrating transcriptome profiles from differential expression analysis of five breeds of beef cattle, 16 circRNAs, 43 lncRNAs, 7 miRNAs, and 237 mRNAs associated with significant biological molecular processes are found
Wang A et al. (2023)		goat	9	transcriptome sequencing	4,404	Intergenic	submandibular gland tissues	Regulate submandibular gland tissues	4,404 lncRNAs were identified from the submandibular gland tissue, with generally low expression levels across different developmental stages of goat submandibular gland
Wei et al. (2023)		yaks	6	RNA-seq analyses	304	Located on chromosome	the longest dorsal muscle	affecting the formation and growth of muscles	LncRNA controlling myogenesis, growth, and development of muscles in Gannan and Jeryak
Huang C et al. (2022)	m^6^A-methylated lncRNAs	Cattle -yak	6	transcriptome sequencing	8,388	Located on chromosome	Longissimus dorsi muscle	encouraging the growth of muscle	Revealed the role of m6A alteration on lncRNA in the conversion of muscle fiber type and muscular growth
Marceau et al. (2022)		Holstein bull	6	RNA-seq analyses	638	Located on chromosome	rumen tissues	Regulate rumen development	Cow performance and rumen development are influenced by rumen lncRNAs, which are only expressed in one of the two weaning settings
Singhal et al. (2022)		Murine	25	microarray	3,195	Unequal distributed with chromosome	Myoblasts cells	regulating muscle cell growth and differentiation	Network analysis and GO keywords point to a potential muscle-specific function for these lncRNAs. Analyzing the pathways of the genes with differential expression reveals their participation in signaling pathways like Hippo, PI3K-Akt, and the control of pluripotency in stem cells
Muniz et al. (2022a)		Nellore cattle	20	RNA-seq analyses	10	located in antisense direction of intronic and exonic regions	Longissimus thoracis muscle	Regulating beef tenderness and marbling traits	Investigating the transcriptional regulatory functions of long noncoding RNAs (lncRNAs) and their growing relevance in the biology of muscles and meat quality attributes
Jara et al. (2022)		Hereford cattle	11	RNA-seq analyses	4,937	Located on chromosome	eyelid skin specimens	Immune Response and Pigmentation	Identified 4,937 potential new lncRNAs and mapped them to the most recent bovine reference genome. Discovered connections between certain lncRNAs and target genes associated with pigmentation, immune responses, and cancer development
Gu et al. (2022)		Hainan black goats	6	RNA-seq analyses	4,396	Located in the cytoplasm and nucleus	Longissimus dorsi muscle	Regulate meat quality	In the Longissimus dorsi muscles of adult Hainan black goats, there is a greater intramuscular fat content, muscle fiber diameter, and shear force when compared to young children. Meat quality is greatly influenced by the genes CPT1A and DGAT2
Tian et al. (2022)		Chicken	6	Ribo-Zero RNA-seq	15,179	Located on chromosome	abdominal adipocytes	Regulating adipogenic differentiation	840 differently expressed lncRNAs were found when the expression patterns and regulatory mechanisms of lncRNAs were examined in chicken adipocytes during differentiation
Yang et al. (2022)		Qinchuan cattle	12	transcriptome sequencing analysis	7,035	Located in the cytoplasm and nucleus	Intramuscular preadipocytes from longissimus dorsi muscle	regulating bovine intramuscular preadipocyte differentiation	31 genes linked to adipogenesis were found using ceRNA mechanism analysis and gene co-expression network analysis
Liu et al. (2022)	LOC104975788	Shandong Black and Luxi Cattle	6	RNA-seq analyses	387	Located on chromosome	Longissimus dorsi muscle	inhibiting the regulation of skeletal muscle development by Pax7 through the action of miR-133a	By competitively suppressing the expression of the target gene Pax7 via bta-miR-133a binding, LOC104975788 controls the development of skeletal muscle
(F. Wang et al., 2022)		Sheep	5	comparative transcriptome analysis	7,848	Located in the cytoplasm and nucleus	liver and lung samples	Regulating hypoxia	Target genes, new long noncoding RNAs, and biological pathways important for altitude adaptation were found
Ye et al. (2022)		Mice	15	RNA-seq analyses	32,105	Located on chromosome	transverse aortic constriction and heart	inhibition of cardiac hypertrophy	LncRNAs may affect genes implicated in the insulin-like growth factor signaling pathway, which may lead to heart hypertrophy in mice, and hence contribute to puerarin and Airn’s cardio protection
Han et al. (2021)	MALAT1	Mouse	60	RNA-seq analyses	1,120	located primarily in the nucleus	Patients with colorectal cancer who underwent surgery	regulating the transcription of PPAR-γ	The suppression of MALAT1 was found to inhibit the process of adipogenesis by controlling the expression of PPAR-γ on a transcriptional level
Chen M et al. (2021)	LNC23	Inner Mongolian black cattle		High throughput sequencing	63,509	localized in nucleus	the liver, spleen, lung, kidney, small intestine, stomach, scapular, gluteus, longissimus, intercostal muscle, and heart	regulated the myogenic differentiation of cattle skeletal muscle	Through the lnc23-PFN1-RhoA/Rac1 axis, the new lnc23 positively regulates the myogenesis of satellite cells in the skeletal muscle of bovine
Jia et al. (2020)		Charolais and Tongjiang cattle	6	RNA-seq analyses	4,155	unequal distribution on chromosomes	bovine longissimus dorsi muscle	organ development, gene regulation and signal transduction	32 lncRNAs were differentially expressed between Charolais and Tongjiang cattle that involved in muscle development
(X. Zhang, M. Chen, Liu et al., 2020)	LNC403	Inner Mongolian black cattle	4	RNA-seq analyses	3	located on bovine chromosome	skeletal muscle	control of KRAS and Myf6 expression to promote the differentiation of cattle satellite cells	The lncRNA known as lnc403 is strongly expressed in the early stages of embryonic development and is unique to the skeletal muscle of cows
Jiang et al. (2020b)		Qinchuan cattle	6	RNA-seq analyses	3,716	mainly located in cytoplasm	subcutaneous adipose tissue	promote fat development	In the adipose tissue of cattle throughout various age groups, lncRNA is significant
(Y. Chen et al., 2020)	lncRNA-Adi	Rat	24	RNA- sequence analysis		located in the cytoplasm	Inguinal fat tissue	Regulate cell division and PPARγ expression during adipogenesis	lncRNA-Adi interacts with miR-449a to enhance the CDK6-pRb-E2F1 pathway, promoting cell proliferation in adipose-derived stem cells. This interaction also affects PPARγ expression during adipogenic differentiation
Cai et al. (2020)	BADLNCR1	Bovine	20	CHIRP-seq	3,339	primarily localized in the nucleus	inguinal fat tissue	inhibits terminal adipocyte differentiation	KLF2 regulates a long non-coding RNA, and BADLNCR1 seems to act as a suppressor in bovine adipocyte differentiation

Researchers are examining how lncRNAs regulate the process of adipogenesis in beef cattle by leveraging genomics (Wei et al., 2016). Recent research has uncovered the intricate epigenetic control of lncRNAs in beef cattle during adipogenesis (Kang et al., 2020). Epigenetic alterations such as DNA methylation and histone modifications play a vital role in regulating the production of lncRNAs that impact adipocyte development and lipid metabolism (Yu et al., 2020a; Mu et al., 2022). The regulation of adipogenesis in beef cattle is significantly influenced by lncRNAs. Several noteworthy long non-coding RNAs linked to this mechanism are HOTAIR, ADNCR, AGAP2-AS1, ANRIL, NEAT1, and MALAT1 (Kang et al., 2020; Chen X et al., 2020; Guo et al., 2021). Academia and cattle farmers could potentially enhance the traits of beef cattle’s growth and meat by employing innovative breeding methods and effective management strategies to understand the epigenetic control of lncRNAs (Ibeagha-Awemu and Zhao, 2015; Zhang et al., 2019). lncRNAs are known to dramatically affect adipogenesis through the process of intramuscular adipocyte development in beef cattle. In addition to controlling gene expression, they support several biological processes linked to adipogenesis. Numerous lncRNAs, such as BIANCR (Ma X et al., 2023) lncBNIP3 (Zhang W et al., 2023), and lnc210 (Zhu et al., 2023), among others, have been identified and studied for their roles in intramuscular adipogenesis. These lncRNAs have been demonstrated to affect adipocyte differentiation, apoptosis, and proliferation via a variety of signaling pathways, including the ERK1/2 pathway (Ma X et al., 2023), The processes connected to DNA replication and the cell cycle (Ru et al., 2023). Furthermore, researching the lncRNAs ' epigenetic regulation in the adipogenesis of beef cattle may have broader implications in understanding the regulation of adipocyte development in other species and potentially in human adipogenesis as well (Zhai et al., 2021). Overall, improving meat quality, strengthening genetic selection, and comprehending adipocyte development in diverse species are all greatly impacted by the study of epigenetic control of long non-coding RNAs within beef cattle adipogenesis (Mu et al., 2022). Moreover, the identification of key lncRNAs involved in adipogenesis opens up new opportunities for targeted manipulation of beef cattle’s fat composition and deposition Zhang et al. (2019) (Nolte et al., 2019; Zhai et al., 2021) (Table 2).

### 5.1 Characterization of lncRNAs in beef cattle

Exploring lncRNAs in beef cattle is an area with potential for understanding gene regulation and identifying new markers for production traits (Liu et al., 2020). Scientists have made progress in describing lncRNAs involvement in biological mechanisms, revealing their varied functions within cells (Liu et al., 2022). Recent progress in genomics and transcriptomics has enabled researchers to explore the world of extended non-coding RNAs. It is now known that these molecules, which were previously believed to be just transcriptional noise, are crucial for the control of genes (Li H et al., 2020). In beef cattle, studying lncRNAs provides unique opportunities to understand the regulatory mechanisms behind important production traits (Huang et al., 2021; Zhang W et al., 2023).

Understanding how lncRNAs interact with protein-coding genes can help us comprehend how they influence cellular processes, hence influencing the molecular landscape of beef cattle (Nolte et al., 2019; Zhang et al., 2019b). The discovery of lncRNAs as possible biomarkers for production traits offers exciting prospects for breeding programs and animal husbandry practices. Understanding the regulatory impact of these non-coding RNAs could improve the targeted selection for favorable characteristics, leading to an overall enhancement of beef cattle populations (Li Q et al., 2020; Yan et al., 2021).

As research the characterization in this field advances of lncRNAs in beef cattle has potential practical applications in agriculture and animal science (Muniz et al., 2022a). High-throughput sequencing technologies can be used to analyze lncRNAs in beef cattle, allowing for the thorough identification and profiling of lncRNAs in a variety of tissues and physiological conditions (Yan et al., 2021). Researchers can understand the possible roles and regulatory mechanisms of lncRNAs by examining their expression patterns and genomic characteristics (Mu et al., 2022). Integrating data from transcriptomic, epigenomic, and functional studies can provide a holistic knowledge regarding the function of lncRNAs in beef cattle (Zhang S et al., 2021). This approach helps identify lncRNAs -target interactions, assess their impact on gene expression, and elucidate their involvement in biological pathways (Zhang et al., 2019b). Studying the evolutionary conservation and genetic variations of lncRNAs in different cattle breeds can improve our comprehension of their significance in evolution and breeding programs (Li Q et al., 2020; Liu et al., 2022).

Numerous studies have examined the genomic locations, structures, and regulatory functions in beef cattle during different stages of adipogenesis in recent years. Wang et al. identified subpopulations of fibro/adipogenic progenitors enriched for genes involved in adipogenesis and fibrogenesis (Wang L et al., 2023). Yang et al. conducted transcriptome sequencing and analysis of different RNA types during intramuscular preadipocyte differentiation to identify genes related to adipogenesis (Yang et al., 2022). Furthermore, another research by Wang et al. compared bovine perirenal and intramuscular adipocytes which led to the discovery of differences in their respective adipogenic characteristics and lipid metabolism. Another investigation focused on intramuscular adipocyte development across different breeds of cattle with an emphasis on identifying key genes and compounds involved in marbling propensity (Jaborek et al., 2023). These investigations offer important new understandings of the molecular processes influencing beef quality, such as the regulatory networks involved in adipogenesis in beef cattle and the tendency for marbling.

### 5.2 LncRNAs functions in adipogenic differentiation

LncRNAs strongly influence the regulation of adipogenic differentiation (Kang et al., 2020). Their role at various stages of adipogenesis, from preadipocyte commitment to mature adipocyte formation, is increasingly acknowledged (Li et al., 2018; Zhang et al., 2019b). Several noteworthy lncRNAs have been discovered to control important transcription factors like PPARγ and C/EBPα, as well as genes linked to adipogenic differentiation (Zhang T N et al., 2020). Moreover, there is mounting proof suggesting that lncRNAs may also impact adipose tissue function and metabolic balance (Lu et al., 2021). Deciphering the chemical processes by which lncRNAs govern adipogenic differentiation holds great potential for developing innovative therapeutic approaches for obesity and associated metabolic condition (Zhang T N et al., 2020).

The first class of lncRNA to be discovered to be involved in adipogenesis is steroid receptor RNA activator (SRA) (Xu et al., 2010). Adipose tissue SRA binds to PPARγ to increase its transcriptional activity, which in turn promotes the development of 3T3-L1 preadipocytes. SRA can also influence TNF-α signaling pathway, insulin-related signal transduction pathways, and the cell cycle of adipocytes (Xu et al., 2010). Obese mice on a high-fat diet showed large increases in SRA expression in their white adipose tissue, whereas SRA knockout animals showed a decrease in fat content. Insulin sensitivity is increased, a large number of adipocyte markers and inflammatory genes are expressed less frequently, and it is resistant to obesity and fatty liver brought on by a high-fat diet (Liu et al., 2014). One family of nuclear long noncoding RNAs that is closely related to the paraspeckle form is called nuclear-enriched transcription factor 1 (NEAT1) (Cooper et al., 2014). NEAT1 contains a binding site for miR-140. Through physiological interactions, mature miR-140 in the nucleus can bind to NEAT1, increasing the latter’s expression. MiR-140 has been shown by Gernapudi et al. to bind to NEAT1 physiologically and upregulate its expression (Gernapudi et al., 2016). Adipogenic differentiation-inducing non-coding RNA (ADINR) is a long non-coding RNA that is situated 450 base pairs upstream of the gene encoding CCAAT/enhancer binding protein α (also known as C/EBPα). When human bone marrow mesenchymal stem cells (MSCs) undergo adipogenic differentiation, its expression is markedly upregulated. While overexpression of C/EBPa can repair poor adipogenesis due to loss of endogenous ADINR, ADINR knockdown has an impact on adipogenesis (Xiao et al., 2015).

The complex role that lncRNAs play in the processes of adipogenic differentiation and metabolic balance highlights the potential to target lncRNAs -mediated regulatory networks for the treatment of obesity and related metabolic diseases (Squillaro et al., 2020). It is crucial to conduct additional investigation into the precise mechanisms and subsequent impacts of lncRNAs participation in adipogenesis to advance successful therapeutic strategies.

LncRNAs are generally well known to be significant modulators of transcription factors and signaling pathways related to the development of adipocytes in cattle reared for meat. Studies have demonstrated that lncRNAs are critical for controlling the ERK1/2 signaling pathway during the adipogenic process (Ma X et al., 2023). Furthermore, different lncRNAs expression patterns have been observed during the intramuscular preadipocyte differentiation process. These patterns are linked to significant noncoding and mRNAs that contribute to the buildup of intramuscular fat (Yang et al., 2022). These findings offer important new insights into the molecular mechanisms governing lncRNAs function across different breeds of cattle. This information may help molecular breeding programs for beef cattle and enhance the quality of beef meat (Liu et al., 2020; Muniz et al., 2022a).

### 5.3 The interaction of lncRNAs and epigenetic regulation in adipogenesis in beef cattle

A number of investigations have shown that specific long non-coding RNAs affect the expression of crucial genes involved in the development of fat cells, controlling the growth of fat cells in cattle (Wang L et al., 2020; Lu et al., 2021). This impact is exerted through interactions with chromatin modifiers, such as histone methyltransferases and DNA methyltransferases. They can also control the expression of microRNAs involved in adipogenesis by functioning like rivaling ceRNAs (Zhang et al., 2023).

Recent studies have also identified specific lncRNAs that are differentially expressed during different stages of adipogenesis in beef cattle, suggesting their active function in controlling the growth of fat cells (Zhang et al., 2019). According to the research, lncRNAs are involved in both the initial creation of fat cells and the maintenance and regulation of adipose tissue (Romao et al., 2014).

The interaction between lncRNAs and epigenetic modifiers in beef cattle adipogenesis reveals the complex regulatory network governing this biological process (Chen et al., 2020). Understanding how lncRNAs affect adipogenesis provides insight into fat cell development and potential implications for enhancing beef production efficiency and meat quality (Guo et al., 2017). Studies in this field indicate that lncRNAs are crucial for the adipogenesis of beef cattle’s epigenetic regulation. Further investigation into their roles and interactions with transcription factors and chromatin modifiers is necessary to gain a thorough understanding of the growth of fat tissue in beef cattle (Zhang et al., 2019; Pant et al., 2021).

Understanding how the epigenetic control of adipogenesis in beef cattle is influenced by lncRNAs presents significant opportunities for creating novel approaches to control the accumulation of fat and enhance meat quality (Jiang et al., 2020b; Chen M et al., 2021). Further research in this area could lead to identifying lncRNAs -based biomarkers for beef cattle adipogenesis and developing lncRNAs -targeted interventions to modulate fat accumulation in beef cattle.

### 5.4 Interplay between lncRNA and environmental factors in beef cattle

LncRNAs and environmental factors are intricately linked, as recent studies in beef cattle have revealed (Zou et al., 2023). When cattle are exposed to environmental stressors such temperature swings, nutritional modifications, and pathogens, their expression is dynamically regulated, indicating a major function for lncRNAs in bovine adaptability (Zeng et al., 2023).

Environmental influences can affect the expression and functionality of lncRNAs in metabolic tissues, including muscle, liver, and adipose tissue (Wang L et al., 2021). For instance, studies show that changes in the surrounding temperature can affect the expression of particular lncRNAs linked to energy metabolism and thermoregulation in beef cattle (Marceau et al., 2022). Dietary changes, like adding heat-stabilized RB or B. longum-FRB to the diet, can affect lncRNAs expression related to nutrient metabolism and gut health (Li Q et al., 2022). Pathogen exposure can also change lncRNAs expression linked to immune response pathways and inflammation in beef cattle (Shen et al., 2023).

Furthermore, environmental factors may significantly affect lncRNAs in beef cattle, impacting health, productivity, and resilience (Hu et al., 2022). Understanding the interaction between lncRNAs and environmental cues holds promise for developing interventions to optimize cattle welfare and production outcomes (Zhang R et al., 2021). Ongoing investigations into these interactions are pivotal for advancing sustainable livestock management and genetic improvement strategies. This study improves our understanding of muscle development regulation in cattle by identifying specific lncRNAs and their regulatory relationships with protein-coding genes (Huang et al., 2021). This realization may open up new avenues for raising the beef cattle’s productivity and the quality of their meat.

**FIGURE 3:**
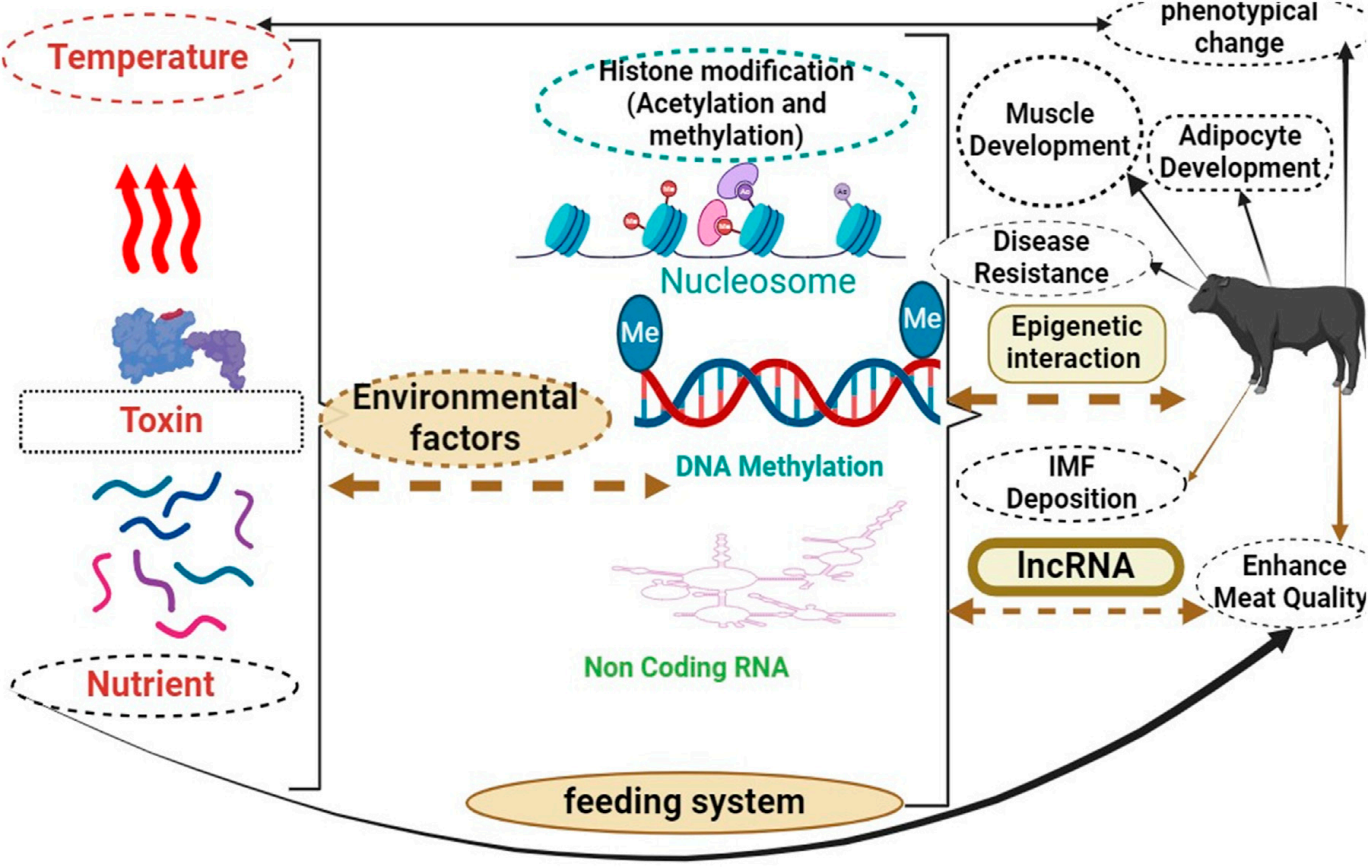
Relationships between environmental factors, lncRNAs, and epigenetics in beef cattle.

Interaction between lncRNAs, environmental factors, and epigenetics in beef cattle is depicted in Figure 3. Arrows connect these procedures with specific regions “Epigenetics”, “lncRNAs,” and “environmental factors” are important in beef cattle biology and muscle development. Understanding their interaction is essential for improving breeding strategies and economic benefits in the industry. Research on these factors can optimize cattle growth and genetic selection methods, leading to improved productivity. Studying their interplay can provide valuable insights into how they influence muscle development and overall animal health. Researchers may identify key molecular mechanisms contributing to muscle development by unraveling this complex relationship.

## 6 Techniques and experimental perspectives

The field of epigenetics is making strides toward comprehending the control of gene expression in beef cattle. Within this regulatory network, significant influence is exerted by lncRNAs on chromatin structure and gene expression (Yue et al., 2019; Wang X et al., 2023). Identification, characterization, and the relationship between particular lncRNAs and epigenetic mechanisms in beef cattle have been the main research areas (Wen et al., 2020).

To find out more about lncRNAs ' functional roles, binding patterns, and levels of expression, researchers studying lncRNAs and epigenetic control in beef cattle employ RNA sequencing, chromatin immunoprecipitation, and CRISPR-Cas9 gene editing technology (Jiang et al., 2020b; Zhao et al., 2020). These techniques offer valuable methods to investigate the function of lncRNAs and epigenetic processes in beef cattle, offering a more comprehensive comprehension of intricate regulatory networks influencing characteristics including growth, procreation, and resistance to disease (Zhao et al., 2020). Combining RNA sequencing, chromatin immunoprecipitation, and CRISPR-Cas9 gene editing offers researchers a comprehensive understanding of the roles played by lncRNAs and epigenetic control in beef cattle, which may reveal new avenues for enhancing livestock industry productivity and health (Jia et al., 2020; Jiang et al., 2020b). Furthermore, employing epigenomic profiling methods like ChIP-seq and ATAC-seq can yield insights into alterations in chromatin accessibility and histone modifications linked to the regulation of lncRNAs within beef cattle (Jiang et al., 2020b; Zhang R et al., 2021). All things considered, these advanced methodologies and research vantage points are essential for illuminating the subtleties of lncRNAs-mediated epigenetic control over beef cattle.

### 6.1 Epigenomic profiling

Understanding the function and regulation of lncRNAs and epigenetic mechanisms, epigenomic profiling techniques are essential (Li, 2021). These methods offer insights into the epigenetic modifications, chromatin structure, and regulatory elements associated with lncRNAs, aiding researchers in uncovering their regulatory networks and roles (Ma and Zhang, 2020). By using specific methods like chromatin immunoprecipitation sequencing and genome-wide DNA methylation analysis, researchers can reveal the epigenetic modifications and histone marks associated with lncRNAs loci (Statello et al., 2021). Additionally, techniques such as RNA sequencing and ribosome profiling help identify lncRNA transcripts and their associations with gene expression profiles, giving more insight into the functional roles they play in biological processes (Zhu et al., 2021).

Technological developments in single-cell sequencing have made it possible to investigate lncRNAs expression and epigenetic modifications demonstrating the dynamic nature of lncRNAs regulation and cellular heterogeneity at the single-cell level (Xu et al., 2023).

### 6.2 RNA sequencing and chromatin immunoprecipitation

RNA sequencing and chromatin immunoprecipitation methods have been widely used to study lncRNAs and epigenetic control in beef cattle (Muniz et al., 2022a). These methods allow the examination of transcription factor and chromatin modifier binding, DNA methylation patterns, histone modifications found to be regulated by lncRNAs, expression patterns, and chromatin modifications linked to lncRNAs (Zhao et al., 2020; Hu et al., 2022). These techniques provide important new understandings of lncRNAs regulatory mechanisms and their effects on beef cattle epigenetic control (Zhang et al., 2022). Through the integration of RNA sequencing and chromatin immunoprecipitation methodologies, scholars can acquire a thorough comprehension of the relationship between lncRNAs and epigenetic regulation, providing insight into significant molecular mechanisms like gene expression, histone modifications, and chromatin dynamics in the development and expansion of beef cattle’s skeletal muscles (Peng et al., 2023). RNA sequencing is the extraction of individual RNA molecules from tissue samples taken from beef cattle, followed by cDNA library conversion and the generation of millions of short reads using next-generation sequencing technologies (Fang et al., 2019).

Prior to chromatin studies, In order to identify, these readings are mapped to a reference transcriptome or genome and quantify various lncRNAs in beef cattle (Marceau et al., 2022). Chromatin immunoprecipitation is a technique used to study the interactions between lncRNAs and epigenetic markers or chromatin-modifying proteins (Li Q et al., 2022). It involves cross-linking and shearing of chromatin, followed by immunoprecipitation using specific antibodies (Marceau et al., 2022; Muniz et al., 2022a). The isolated DNA is then sequenced and aligned to identify the genomic regions bound by the target protein or modified with the specific epigenetic marker (J. Wang et al., 2022). The technique offers understanding of the relationships between long non-coding In epigenetic regulation, the functional roles of RNAs and chromatin modifiers (Alexandre et al., 2020). Scientists use RNA sequencing and chromatin immunoprecipitation to study the regulatory mechanisms and Functional importance of extended noncoding RNAs (Li Y et al., 2020). Planning suitable experiments and employing specific methods are essential for studying lncRNAs and epigenetic regulation in beef cattle.

## 7 Developing targeted breeding strategies for improving meat quality

There exists substantial genetic diversity in traits related to growth, carcass, and meat properties that can be utilized for selective breeding in animal breeding programs. It is crucial to establish tailored breeding methods to improve meat quality. A primary strategy for enhancing meat quality through breeding involves selectively choosing specific desirable characteristics (Abd El-Hack et al., 2018). These attributes may encompass marbling, tenderness, flavor, and overall muscle composition. By emphasizing these particular traits, breeders can strive to cultivate animals that consistently yield superior-quality meat (Svitáková et al., 2014). Selective breeding is important, but it is also crucial to take genetics into account when looking at meat quality. Identifying genetic markers linked to better meat quality can help choose animals with the most potential for passing on these desirable traits to their offspring (Zalewska et al., 2021). Additionally, the incorporation of advanced technologies like genomic selection can offer a more extensive insight into the genetic structure responsible for meat quality characteristics. This allows breeders to make better-informed choices when selecting breeding animals, ultimately expediting enhancements in meat quality within livestock populations (Stock and Reents, 2013; Bedhane et al., 2019). It is also crucial to take into account environmental aspects that may affect meat quality, as they can interact with genetic factors. By adopting a comprehensive approach that acknowledges both genetic and environmental elements, breeding methods can be fine-tuned to consistently yield animals with exceptional meat-quality attributes (Leal-Gutiérrez et al., 2020; Del Campo et al., 2021; Li Y et al., 2022). In order to enhance the quality of meat, it is essential to employ a comprehensive strategy that involves selective breeding, genetic knowledge, technological progress, and environmental factors. By combining these components effectively, breeders can make significant progress in improving the overall quality of meat within livestock populations (Tonussi et al., 2015; Abd El-Hack et al., 2018).

## 8 Challenges and future directions

The complex interplay between lncRNAs and epigenetic processes during beef cattle adipogenesis poses difficulties in pinpointing particular lncRNAs and comprehending the intricate nature of epigenetic control. The multifaceted interaction among genetic, environmental, and nutritional elements, along with breed-specific variations, hinders the accurate understanding of these regulatory systems. Unraveling how lncRNAs contribute to in beef cattle, adipogenesis and meat quality features necessitate comprehensive approaches combining transcriptomics, epigenomics, and functional genomics. Identifying specific lncRNAs involved in adipogenesis is also a challenge due to limited comprehension of their operations and modes of operation. More research on the functions of lncRNAs in adipogenesis and characteristics of beef cattle’s meat quality will require advanced technologies, such as next-generation sequencing, to accurately identify and characterize lncRNAs.

Despite these difficulties, upcoming developments in genomic technologies like single-cell sequencing and CRISPR-based gene editing provide accuracy in pinpointing important contributors to adipogenesis and meat quality traits. These advancements will aid in unraveling the complex regulatory networks involving lncRNAs and epigenetic modifications.

Integrating different kinds of omics data, like as transcriptomics, epigenomics, and metabolomics, can lead to a thorough understanding of the mechanisms underlying adipogenesis and meat quality. Integrating multi-omics data with big data analytics and machine learning has the potential to reveal intricate patterns within this complex network. This will advance our knowledge of how lncRNAs and epigenetics affect adipogenesis and characteristics of meat quality, improving both consumer satisfaction and production efficiency. Comparative research spanning various species and the incorporation of these insights into breeding programs not only improves beef quality but also addresses broader implications such as conditions related to obesity. The coming together of technological advances and interdisciplinary approaches presents a positive prospect for understanding the complexities surrounding lncRNAs -epigenetic interactions linked to adipogenesis in beef cattle.

## 9 Conclusion

In conclusion, this review has highlighted the complex process that use lncRNAs and epigenetic mechanisms to control the formation of adipocytes in beef cattle. Enhancing meat quality, molecular breeding techniques, and research on metabolic disorders in the beef industry can be greatly aided by the several lncRNAs involved in adipogenesis have been identified, and their effects on gene expression and subsequent biological processes have been studied. Contemporary genomic advancements, such as molecular markers and genetic variations, can improve animal productivity and address the growing global need for premium meat. This review provides critical information for the field of epigenetics by demonstrating the critical role lncRNAs play in regulating gene expression and adipogenic biological processes. lncRNAs have been identified and validated as potential biomarkers, which offers new opportunities to advance the creation of therapeutic or diagnostic interventions aimed at enhancing the characteristics of beef cattle’s meat quality. An integration of lncRNAs research with other omics technologies and genetic selection strategies has the potential to significantly improve the beef industry by guiding breeding programs for better meat quality and production efficiency, benefiting both producers and consumers. This thorough integration of existing information provides a basis for future research endeavors focused on understanding the intricate connections between lncRNAs and epigenetics in adipogenesis in beef cattle. This work will help advance scientific understanding and practical applications in animal agriculture.

## Abbreviations

AP-1, Activator Protein-1; ceRNAs, Competitive Endogenous RNAs; DNMT, DNA Methyl Transferases; DLK1, Delta-Like 1 Homolog; FABP4, Fatty Acid-Binding Protein 4; IGF2R, Insulin Growth Factor 2 Receptor; IMF, Intra Muscular Fat; LncRNAs, Long Noncoding RNAs; LPL, Lipoprotein Lipase; MAPK, Mitogen-Activated Protein Kinases; MSCs, Mesenchymal Stem Cells; PPARG, Peroxisome Proliferator-Activated Receptor Gamma; PPARγ, Peroxisome Proliferator-Activated Receptor Alpha; RXRA, Denotes Retinoic X Receptor Alpha.
